# Estimating the Relative Impact of Clinical and Preventive Community-Based Interventions: An Example Based on the Community Transformation Grant Program

**DOI:** 10.5888/pcd16.180594

**Published:** 2019-07-03

**Authors:** Benjamin Yarnoff, Christina Bradley, Amanda A. Honeycutt, Robin E. Soler, Diane Orenstein

**Affiliations:** 1 RTI International, Research Triangle Park, North Carolina; 2 Centers for Disease Control and Prevention, Atlanta, Georgia

## Abstract

**Introduction:**

Public health focuses on a range of evidence-based approaches for addressing chronic conditions, from individual-level clinical interventions to broader changes in policies and environments that protect people’s health and make healthy living easier. This study examined the potential long-term impact of clinical and community interventions as they were implemented by Community Transformation Grant (CTG) program awardees.

**Methods:**

We used the Prevention Impacts Simulation Model, a system dynamics model of cardiovascular disease prevention, to simulate the potential 10-year and 25-year impact of clinical and community interventions implemented by 32 communities receiving a CTG program award, assuming that program interventions were sustained during these periods.

**Results:**

Sustained clinical interventions implemented by CTG awardees could potentially avert more than 36,000 premature deaths and $3.2 billion in discounted direct medical costs (2017 US dollars) over 10 years and 109,000 premature deaths and $8.1 billion in discounted medical costs over 25 years. Sustained community interventions could avert more than 24,000 premature deaths and $3.4 billion in discounted direct medical costs over 10 years and 88,000 premature deaths and $9.1 billion in discounted direct medical costs over 25 years. CTG clinical activities had cost-effectiveness of $302,000 per death averted at the 10-year mark and $188,000 per death averted at the 25-year mark. Community interventions had cost-effectiveness of $169,000 and $57,000 per death averted at the 10- and 25-year marks, respectively.

**Conclusion:**

Clinical interventions have the potential to avert more premature deaths than community interventions. However, community interventions, if sustained over the long term, have better cost-effectiveness.

**Summary**

**What is already known on this topic?**

Previous work demonstrated the potential long-term impact of clinical and community interventions to prevent chronic disease. However, that work considered only hypothetical interventions that may not accurately reflect the feasibility of implementation in a real-world setting.

**What is added by this report?**

We examined the potential 10- and 25-year impact of clinical and community interventions to prevent chronic disease as they were implemented under the Community Transformation Grant program.

**What are the implications for public health practice?**

Results support public health practitioners in strategic planning for chronic disease prevention.

## Introduction

Public and private sector stakeholders have worked together for decades to prevent chronic disease, improve quality of life, and reduce medical costs and death associated with chronic disease. Evidence-based approaches for addressing chronic conditions range from individual-level clinical interventions addressing better identification and control of chronic diseases to broader changes in policies and environments around diet, physical activity, and smoking that make healthy living easier in a community. Public health now focuses on all these areas but recognizes that different interventions may have different potential impacts (1). Assessing the potential impact of interventions is challenging, because interventions take time to affect health and economic outcomes. As a result, only a small part of the impact of these interventions can be quantified in the first few years through observing program reach and initial impact on behaviors. Simulation modeling is a useful tool to extend the time horizon for assessing the potential long-term impact of clinical and community interventions.

Previous comparisons of clinical and community interventions generally considered policy change scenarios that may not have accurately reflected the real-world applications of these interventions (2). In this study, we simulated the potential 10- and 25-year impacts of 2 types of interventions as they were implemented as part of the Community Transformation Grant (CTG) program, a large multicommunity public health program funded by the Centers for Disease Control and Prevention (CDC) from 2011 through 2014.

## Methods

The CTG program is a large-scale example of a program that supported the implementation of both clinical and community approaches to address chronic disease (3). CTG awardees were required to address at least one of the following focus areas: 1) increase options for tobacco-free living (eg, smoke-free policies for workplaces or multiunit housing), 2) promote and improve access to opportunities for active living and healthy eating (eg, working with partners to build bike paths and increase the availability of fruits and vegetables at corner stores), 3) increase use of clinical and community preventive services (eg, community health worker initiatives), and/or 4) expand access to healthy and safe physical environments (eg, Safe Streets initiatives) (4). After a competitive application process, CDC allocated $103 million to 61 state and local government agencies, tribes and territories, and nonprofit organizations in 36 states, covering 130 million people (3,5).

We used the CTG program as an example of a chronic disease prevention program to estimate the long-term potential health and economic outcomes of clinical and community interventions if they were sustained at the same level over time. We used information on the classifications of interventions that were conducted as part of the CTG program and their reach as inputs to the Prevention Impacts Simulation Model (PRISM) to estimate the potential long-term impact of clinical and community interventions. In the CTG program, reach was operationalized as the estimated number of people in the target population who had increased access to (eg, those living within 1 mile of a park), are protected by (eg, a workplace smoke-free policy), or are otherwise affected by (eg, patients covered by a community health worker program) an intervention (6).

PRISM is a computer simulation model containing mathematical equations that describe how risk factors interact to produce chronic disease and poor health outcomes and the impacts of various community and clinical interventions. PRISM calculates outcomes annually and cumulatively from 1990 through 2040 (7–10). PRISM was validated in several ways during its development and has been used to estimate the long-term impact of other community health programs, such as the Communities Putting Prevention to Work program (11) and public health prevention activities of the Los Angeles County Public Health Department (12).

PRISM includes a wide range of chronic disease–related intermediate outcomes that can be influenced by clinical and community intervention strategies. These strategies are represented in the model as “levers,” which reflect changes in the numbers of people reached by the strategy. Lever movement provides an estimate of the intent-to-treat population and not the population that changed their health behaviors as a result of lever movement. PRISM simulates the impact of lever movement on cardiovascular disease (CVD) risk behaviors, like smoking and physical activity in the reached population, by applying published estimates of the effect of increased access on health behavior. For example, building a park would increase the lever for access to physical activity spaces; PRISM then simulates the impact on physical activity for the portion of the reached population that used the park and increased their physical activity. These impacts on risk factors, in turn, reduce the prevalence of cardiovascular disease, pulmonary disease, lung cancer, and resulting deaths and costs. PRISM includes levers that address tobacco use; nutrition; physical activity; clinical care for preventing or mitigating hypertension, diabetes, and high cholesterol; and aspirin use. Most PRISM levers are represented by an index ranging from 0 (no implementation of the strategy) to 1 (optimal implementation of the strategy across the entire population). Because PRISM levers represent broad strategies to improve access, each PRISM lever can be moved by one or more evidence-based interventions. For example, the lever “Increasing access to physical activity spaces” can be moved by each of 10 interventions that are expected to produce a positive health outcome, including bike shares (13–17), safe-streets initiatives (18,19), parks (19-21), and joint-use agreements (22–24). Each evidence-based intervention was assigned to an intensity category (minimal, low, medium, and high) that represented its ability to move the lever for those reached by the intervention. The intensity category was assigned primarily on the basis of the impact of the intervention estimated in the literature. A list of all evidence-based interventions that can move each lever and details on the process of generating the list and assigning intensity categories are available in an online supplement (https://forio.com/app/cdc/prism/#/resources).

PRISM simulation outcomes reflect the impact of changes in lever settings compared with baseline trends (ie, no change from the status quo). Baseline PRISM levers were set to reflect a community’s public health environment pre-intervention (ie, before the CTG program began, in 2011). For example, when analyzing the impact of increasing access to physical activity spaces, we did not simply assume that a community started from a baseline access level of zero, but instead we used publicly available information about each community’s policies and environment to estimate the baseline level for each lever. Baseline lever settings were determined by reviewing data and literature on the existing environment for physical activity, nutrition, tobacco, and clinical services policies, such as city, county, and state information from the literature, and secondary data sources, such as the US Census Bureau and the National Health and Nutrition Examination Survey.

### Translating CTG activities into PRISM inputs

Building on previous work (11), we used the RE-AIM (reach, effectiveness, adoption, implementation, maintenance) framework to translate CTG activities into PRISM lever inputs for simulation modeling (25–27). The evaluation focused on reach and effectiveness. To assess reach, we used awardee-submitted estimates of the number of people reached by their activities. CDC provided awardees with written guidance on estimating reach, including metrics, definitions, and potential data sources. Awardees were also encouraged to obtain technical assistance from CDC project officers when estimating intervention reach. Reach was operationalized as the estimated number of people in the target population who had increased access to (eg, those living within 1 mile of a park), are protected by (eg, a workplace smoke-free policy), or affected by (eg, patients covered by a community health worker program) an intervention (6). Determining reach included 1) documenting the setting where the intervention was implemented during the funding period, 2) using census data or setting-specific data (eg, school enrollment) to identify the population count for the setting where the intervention was implemented, and 3) aggregating data. If interventions were implemented in settings or populations that potentially overlapped, the overlap was estimated and accounted for in the aggregation process. Submitted reach estimates were reviewed and validated by trained CDC program officers, subject matter experts, and contractors by using census, school enrollment, and other local data sources.

Because reach was an intent-to-treat metric, not all people reached by the intervention will use the intervention or change their behavior as a result of access. The model incorporates effect-size estimates for the proportion reached whose use and behavior changes (ie, the estimated proportion of people in the target population who have increased access to or are protected by an intervention). Because PRISM is a population model representing the entire community, the denominator for proportional reach was the entire adult population, child population, or the total population of the targeted community as indicated by the US Census Bureau.

To assess effectiveness, we used information on the interventions completed by each awardee as reported in the annual reports submitted to CDC. A team of coders reviewed each awardee’s progress reports and determined which evidence-based interventions (https://forio.com/app/cdc/prism/#/resources) were conducted as part of each awardee activity. Each evidence-based intervention was assigned a categorical intensity that was used to determine the PRISM lever movement. For 20% of the awardee activities, a second coder performed a secondary review for quality control, and the 2 coders reconciled differences.

We computed the lever movement for each activity by taking the intensity of the interventions conducted as part of that awardee’s activity and multiplying by proportional reach. We then computed the total lever movement for each awardee by aggregating the lever movements for all of that awardee’s activities that affected each lever.

We estimated the impact of a subset of CTG activities that met our criteria for being evidence-based on premature deaths averted and medical costs saved after 10 and 25 years. The goal of the CTG program was to implement clinical and community interventions that could be sustained into the future with minimal further input, so we assumed that all interventions would be sustained at a constant level and that maintenance costs would be incurred for at least 10 and 25 years. We also examined the projected program implementation costs of awardee activities (including program maintenance costs) and the projected impact on risk factor management costs to calculate the total cost and cost-effectiveness of the CTG program. We constructed cost-effectiveness ratios as the sum of implementation costs and net medical costs (ie, risk factor management costs minus medical cost savings) divided by the incremental health gains of the program (ie, premature deaths prevented). We estimated the impact of each awardee’s activities overall and separately for clinical and community levers. We examined the median and range of the estimated impact across awardees and the aggregate for all CTG awardees. Medical costs were inflated to 2017 dollars by using the medical cost component of the Consumer Price Index (28). Future cost savings were discounted by 3% per year (29).

We conducted a probabilistic sensitivity analysis in which model parameters were varied across a distribution assumed on the basis of the literature (29) to estimate the lower and upper bounds of a 95% confidence interval for premature deaths averted, risk factor management costs, medical costs saved, and cost per premature death averted.

## Results

Of the 61 CTG program awardees, 29 worked to build capacity for public health interventions and did not implement any interventions. The remaining 32 awardees implemented interventions that could be translated into PRISM levers and were included in this analysis. These awardees covered a population of 87 million people. They implemented clinical interventions reaching 19 million people, community tobacco interventions reaching 20 million people, community nutrition interventions reaching 37 million people, and community physical activity interventions reaching 26 million people.

CTG awardees worked on interventions that affected 21 different PRISM levers (Table 1). Thirty awardees worked on interventions targeting community PRISM levers (including nutrition, physical activity, and tobacco) and 12 awardees worked on interventions targeting clinical PRISM levers. Physical activity access was the lever addressed by the largest number of CTG awardees (20 awardees) and was increased an average of 20 percentage points across all awardees (ie, a 20 percentage-point increase in the number of people with access to places where they can engage in physical activity). Smoke-free multiunit housing was implemented by 18 awardees, with an average movement of 10 percentage points (ie, a 10 percentage-point decrease in multiunit housing complexes that permit smoking). Other levers moved in our analysis were fruit and vegetable access (12 awardees, average movement = 12 percentage points), physical activity promotion (15 awardees, average movement = 7 percentage points), physical activity requirements in schools (13 awardees, average movement = 11 percentage points), and workplace smoke-free policy (12 awardees, average movement = 23 percentage points). The most frequently implemented clinical interventions were related to improving quality care for people with diabetes (8 awardees, average movement = 12 percentage points), hypertension (7 awardees, average movement = 8 percentage points), and high cholesterol (11 awardees, average movement = 7 percentage points).

**Table 1:** Summary of PRISM Levers Moved as a Result of the Community Transformation Grant Program, Number of Communities that Moved Each Lever, and Average Movement of Levers^a^

PRISM Lever	Description of Lever	No. of Communities Moving the Lever	Average Lever Movement, Percentage Points^b^
**Community lever**			
Fruit and vegetable access	The percentage of the population having convenient, affordable access to fresh fruits and vegetables.	12	12
Fruit and vegetable promotion	The extent of promotion for fruit and vegetable consumption through local communication and food placement in the locations in which people typically buy or consume food, as well as through mass media.	4	2
Physical activity access	The percentage of adults with access to safe and affordable walking, biking, social, and green space opportunities for physical activity in worksites and community locations.	20	20
Physical activity promotion	The extent of local communication, placement, and pricing of physical activity options at worksites and in the community, as well as use of mass media and social marketing.	15	7
Physical activity requirements in childcare	The percentage of children aged 2 to 5 in daily childcare that is required to meet recommended physical activity levels and not to exceed screen time limits.	4	3
Physical activity requirements in schools	The percentage of children aged 6 to 17 that is required to meet recommended physical activity levels during school or in after-school programs.	13	11
Smoke-free multiunit housing	The percentage of multiunit housing residents that live in housing that allows smoking.	18	10
Smoke quit services	The use of smoking quit services as affected by affordability, availability, and outreach.	9	24
Smoking counter marketing	Local communication about tobacco products in locations where people shop, work, and live, as well as a mass media social marketing campaign.	5	4
Workplace smoke-free policies	The percentage of indoor workplaces, including restaurants and bars, that allow smoking.	12	23
**Clinical lever**			
Use of quality CVD care after a CVD event	The percentage of the post-CVD population receiving cardiovascular care according to current clinical practice guidelines.	1	4
Use of quality diabetes care non-CVD	The percentage of the non-CVD/post-CVD population with diagnosed diabetes that is receiving diabetes care according to current clinical practice guidelines.	8	12
Use of quality diabetes care after a CVD event		7	12
Use of quality high cholesterol care non-CVD	The percentage of the non-CVD/post-CVD population with diagnosed high cholesterol that is receiving cholesterol care according to current clinical practice guidelines.	11	7
Use of quality high cholesterol care after a CVD event		10	7
Use of quality hypertension care non-CVD	The percentage of the non-CVD/post-CVD population with diagnosed hypertension that is receiving hypertension care according to current clinical practice guidelines.	7	8
Use of quality hypertension care after a CVD event		7	8
Aspirin use compliance female, aged <65	The percentage of prophylactic (daily or every other day) aspirin use among the target population for whom such use is recommended by the US Preventive Services Task Force.	1	1
Aspirin use compliance, female, aged ≥65		1	1
Aspirin use compliance, male, aged <65		1	1
Aspirin use compliance, male, aged ≥65		1	1

Abbreviation: CVD, cardiovascular disease; PRISM, Prevention Impacts Simulation Model.

a PRISM is a computer simulation model containing mathematical equations that describe how risk factors interact to produce chronic disease and poor health outcomes and the impacts of various community and clinical interventions (7–10). Clinical and community intervention strategies are represented in the model as “levers,” which reflect changes in the numbers of people reached by the strategy.

b Movement is defined as an improvement from the baseline lever level (ie, percentage-point change from baseline). Movement reflects only changes in the fraction of the targeted population that had increased access and does not reflect the percentage of people that changed behavior as a result of increases in the levers.

Results from PRISM simulations indicate that the projected 10-year impact (from 2015 through 2024) of clinical levers moved by CTG awardee activities would be more than 36,000 premature deaths averted, $3.2 billion in discounted medical cost savings, and $14.2 billion in risk factor management costs incurred (Table 2). The projected 10-year impact of community levers moved by CTG awardee activities would be nearly 25,000 premature deaths averted, $3.4 billion in discounted medical cost savings, and $3.0 billion in risk factor management costs incurred. The 10-year cost-effectiveness of CTG clinical activities was $302,000 per premature death prevented. The estimated cost-effectiveness of CTG community activities was $169,000 per premature death prevented.

**Table 2:** Projected 10-Year and 25-Year Cost-Effectiveness of Community Transformation Grant (CTG) Activities for Clinical and Community Levers^a^

Outcome	Clinical Levers (N= 12)	Community Levers (N= 30)
**Projected 10-year cost-effectiveness**		
Premature deaths averted	36,530 (35,169–37,730)	24,486 (13,942–41,164)
CTG program implementation costs, $, billion	0.1 (0.1–0.1)	4.6 (3.9–5.3)
Discounted medical cost savings, $, billion	3.2 (3.0–3.4)	3.4 (2.2–5.5)
Risk factor management costs incurred, $, billion	14.2 (11.6–16.1)	3.0 (3.0–3.0)
Total costs,^b^ $, billion	11.0 (8.3–13.2)	4.1 (2.8–4.8)
Cost per premature death averted,^c^ $	302,000 (220,000–374,000)	169,000 (68,000–342,000)
**Projected 25-year cost-effectiveness**		
Premature deaths averted	109,130 (104,850–113,180)	88,374 (51,315–140,496)
CTG program implementation costs, $, billion	0.2 (0.2–0.2)	7.6 (6.4–8.8)
Discounted medical cost savings, $, billion	8.1 (7.6–8.5)	9.1 (5.7–14.3)
Risk factor management costs incurred, $, billion	28.4 (23.2-32.2)	6.5 (5.9–7.5)
Total costs,^b^ $, billion	20.5 (15.0–24.8)	5.0 (2.0–6.7)
Cost per premature death averted,^c^ $	188,000 (132,000–236,000)	57,000 (14,000–130,000)

Abbreviation: CTG, Community Transformation Grant.

a All values are point estimate (lower bound–upper bound).

b Total costs = Program Implementation Costs – Medical Costs Averted + Risk Factor Management Costs Incurred.

c Cost per Death Averted = Total Costs/Deaths Averted.

The projected 25-year impact (from 2015 through 2039) of clinical levers moved by CTG awardee activities would be more than 109,000 premature deaths averted, $8.1 billion in discounted medical cost savings, and $28.4 billion in risk factor management costs incurred (Table 2). The projected 25-year impact of community levers moved by CTG awardee activities would be more than 88,000 premature deaths averted, $9.1 billion in discounted medical cost savings, and $6.5 billion in risk factor management costs incurred. The 25-year effectiveness of CTG clinical activities was $188,000 per premature death averted, and the 25-year effectiveness of CTG community activities was $57,000 per premature death averted.

## Discussion

This analysis provides estimates of the effects of large-scale clinical and community interventions as they were implemented during the CTG program, complementing previous work estimating the impact of hypothetical interventions (2). Results show that CTG clinical activities were projected to avert more premature deaths after 10 years and 25 years than CTG community interventions, but that the gap between the intervention categories shrank from the 10-year mark to the 25-year mark. However, CTG community interventions were projected to save more medical costs after 10 years and 25 years than CTG clinical interventions; this gap increased from the 10-year mark to the 25-year mark. Community interventions in the CTG program had much higher projected program implementation costs than clinical interventions, but led to a much smaller increase in risk factor management costs at the 10-year and 25-year marks. No standard benchmark exists to assess the cost-effectiveness in relation to premature deaths. However, Neumann and colleagues recommended using $100,000 or $150,000 as acceptable amounts to pay per quality-adjusted life year (QALY) gained (30). A cost-effectiveness threshold for premature deaths prevented would be expected to be greater than that for QALYs gained because, on average, preventing a premature death is expected to have a higher value than 1 QALY. Based on this cost-effectiveness threshold, sustained community interventions would likely be considered cost-effective, especially when considered over a period of 10 years or longer.

A previous study using similar methods evaluated another CDC-funded program, Communities Putting Prevention to Work (CPPW), and projected that the program would prevent 14,000 premature deaths in 51 communities during a 10-year period (11). The larger number of premature deaths prevented by the CTG program versus CPPW is likely attributable to the CTG program’s use of clinical interventions, our additional analytic efforts to code evidence-based interventions into PRISM, and the use of existing infrastructure by high-capacity awardees to implement community health interventions.

Our analysis is subject to several limitations. First, all simulation models are approximations to reality and are limited by the evidence of effect sizes that is available. Second, we derived model inputs from awardee progress reports, which may overstate accomplishments. Third, although PRISM is a broad cardiovascular disease model, it accounts for most, but not all, strategies implemented in the CTG program (eg, it does not account for outdoor smoke-free air regulations). Fourth, the analysis assumes that all activities would be sustained for 10 years and 25 years, which is the most optimistic scenario possible. In reality, interventions often lose strength once they are no longer actively promoted. This assumption may be more reasonable for interventions that change policies or the community environment, but may be less realistic for interventions that require regular ongoing support. Fifth, translating programmatic information into any simulation model is challenging, and quantifying community policy and environmental changes introduces aspects of subjectivity. The process used in this analysis was refined from CPPW to reduce subjectivity by focusing on evidence-based interventions from the literature, all of which were assigned to a given category of impact. This approach is consistent with approaches used by others to estimate the “dose” for community health interventions (25–27). Finally, this analysis focused on the aggregate impact of the CTG program and did not address variability in reach and potential health and economic outcomes for specific awardees or target populations.

Study findings suggest that clinical and community interventions, like those implemented in the CTG program, may be expected to have substantial benefits. Clinical interventions have the potential to prevent more premature deaths than community-based interventions in both the intermediate (10 years) and long term (25 years). However, sustaining community-based interventions over the long term may save more in medical costs and have greater cost-effectiveness than investing in only clinical interventions.
